# Do baseline industry and job group skill level predict welfare dependency at 1, 3 and 5 years after mental health related sickness absence? A Danish cohort study

**DOI:** 10.1186/s12889-022-13105-z

**Published:** 2022-04-09

**Authors:** Harald Hannerz, Mari-Ann Flyvholm

**Affiliations:** The National Research Centre for the Working Environment, 105 Lersø Parkallé, DK-2100 Copenhagen, Denmark

**Keywords:** Industry, Occupation, Mental health, Sick leave, Return to work

## Abstract

**Background:**

The cost of mental ill health in the EU-28 nations has been estimated at approximately 4.1% of the total gross domestic products (GDP). Improved rates of return to sustainable employment among people who are sick-listed due to mental ill health would decrease spending on welfare benefits. The present cohort study provides statistical information that may be helpful in the design and prioritizing of efforts aimed at reducing the burden of sickness absence due to mental ill health among employees in the general working population of Denmark. Our primary aim was to estimate odds of being i) deceased or recipient of health related welfare benefits and ii) recipient non-health related welfare benefits, compared to being alive and self-reliant at 1, 3 and 5 years after first visit to a jobs and benefits office due to mental health related sickness absence, as a function of industrial sector and job group skill level at baseline. A secondary aim was to analyze these odds as a function of baseline age, gender, type of mental ill health, family type and employment status.

**Methods:**

The study population consisted of 20–54 year-old persons on long-term sickness absence due to mental health problems in 21 Danish municipalities in 2010–2012 (*N* = 19,660). Odds ratios were estimated by use of multinomial logistic regression. The outcomes were ascertained through national registers.

**Results:**

We did not find any statistically significant association between baseline industrial sector or job group skill level and welfare dependency at follow-up. In the secondary analyses, the estimated odds of health and non-health related welfare dependencies at follow-up tended to increase with unemployment, age, being single and being on sick leave due to self-reported anxiety or depression versus stress/burnout at baseline.

**Conclusions:**

The present study does not support that industry and job group skill level predict welfare dependency after health related sickness absence, after adjustment for relevant covariates, in the general population of Denmark. It suggests, however, that the vulnerability lies in population groups characterized by unemployment, older age, being single and being on sick leave due to self-reported anxiety or depression versus stress/burnout.

## Background

It has been estimated that mental ill health in the EU-28 nations costed a total of approximately 600 billion euro in 2015, which corresponds to 4.1% of the total gross domestic products (GDP) [1]. The estimated costs were divided into i) direct spending on health care (1.3% of GDP), ii) spending on social security programs (1.2% of GDP) and iii) indirect costs due to mental illness related reductions in employment and productivity (1.6% of GDP). The total cost of mental ill health in Denmark was estimated at 14.6 billion euro (5.4% of GDP), which, in terms of percentage of GDP, was the highest estimated cost among the 28 EU-nations.

It is obvious that improved rates of return to sustainable employment among people who are sick-listed due to mental ill health would firstly decrease spending on welfare benefits and secondly decrease costs associated with lost productivity. It has moreover been suggested that employment can improve mental health and that a swift return to sustainable employment thereby also might decrease future health care costs [2, 3]. A lot of time and money have therefore been invested in research aimed at identifying facilitators and barriers of return to work (RTW) among people who are sick-listed due to mental ill health, and at least 11 review articles have been published on the topic [4–14]. With regard to work related factors, there appears to be moderate evidence that social support from supervisors and co-workers are positively correlated with RTW (cf. [6, 7]) and that psychological demands, job strain and exposure to violence and bullying at work are negatively correlated with RTW (cf. [6]). For sociodemographic factors, there are strong evidence for a negative association between age and RTW (cf. [6, 7, 12]). The evidence for an association with RTW is, however, inconsistent for gender (cf. [6, 7]), education (cf. [7]), socioeconomic status (cf. [6, 8]) and marital status/cohabitation (cf. [4, 6]).

The purpose of the present cohort study was to obtain and provide statistical information that may be helpful in the design and prioritizing of efforts aimed at reducing the burden of sickness absence due to mental ill health among employees in the general working population of Denmark. Our primary aim was to estimate odds of being i) deceased or recipient of health related welfare benefits and ii) recipient non-health related welfare benefits, compared to being alive and self-reliant at 1, 3 and 5 years after first visit to a jobs and benefits office due to mental health related sickness absence, as a function of industrial sector and job group skill level at baseline. All analyses would be adjusted for, inter alia, type of mental ill health, gender, age, family type and baseline employment status. Our secondary aim was to calculate odds ratios for welfare dependencies at follow-up also as a function of the above mentioned control variables, which we will refer to as secondary predictors.

### Previous research on the effect of industry and job group on RTW after absence due to mental ill health

It has previously been shown that the incidence of mood disorder as well as the incidence of disability retirement, in the general working population of Denmark, are highly dependent on industrial sector [15, 16] and job group skill level [17, 18]. It was therefore reasonable to believe that these factors may play an important role in the rates of RTW and welfare dependency after onset sickness absence due to mental ill health. Some of the previous research papers on this topic support this supposition while others do not.

#### Re. Industry

In our literature search, we found two large studies that examined the duration of work absence among individuals sick-listed due to mental ill health, by industrial sector. One of the studies concerned sickness absence due to any type of mental ill health in Australia 2005–2007 [19] while the other concerned sickness absence due to depressive symptoms in the Netherlands 2002–2005 [20]. Both of these studies found significant industrial differences and in both of the studies, the education industry was associated with the longest work absence.

#### Re. Job group

Ervasti et al. [8] estimated the rate ratio for RTW after disability due depression at 1.08 (95% CI: 1.03–1.14) for high vs low occupational position among 9908 employees in the Finish Public sector study 2005–2008. The estimate was adjusted for sex, age, and somatic comorbidity.

Ebrahim et al. [21] estimated the rate ratio for RTW after disability due to depression at 0.85 (99% CI: 0.79–0.91) among employees in white (*N* = 2502) versus blue collar industries (*N* = 4532) in Canada 2007–2010.

Engstrom et al. [22] estimated odds ratios for being “not sick” at 2 and 3 years after long-term sickness absence due stress-related psychiatric diagnoses, among 911 employees in Sweden, 2000. They did not find any statistically significant associations with job group (Management, Caring, Education, Service, Other).

Laaksonen and Gould [23] estimated rate ratios for RTW in 2008–2012 among 4297 Finnish residents who were on temporary disability pension (TDP) due to mental disorders in 2008. The rate ratio for Non-manual versus Manual employees was estimated at 1.63 (95% CI: 1.37–1.93) when adjusted for age and at 1.05 (95% CI: 0.87–1.27) when adjusted for age, gender, educational level, employment sector, unemployment before TDP and rehabilitation during TDP.

Nielsen et al. [24] estimated rate ratios for RTW after sickness absence due to mental health problems (MHPs) among 644 employees in Denmark in 2007. They did not find any statistically significant associations with job group (Research, art and technical; Management; Administration; Trade; Service; Manual work; Health care) after adjustment for RTW expectancy, prior absence with MHPs, age, gender and self-reported reason for absence.

Virtanen et al. [25] estimated the association between occupational groups and RTW after a psychiatric work disability period among 3938 public-sector employees in Finland 1997–2005. The occupations were divided into the categories “manual”, “lower level non-manual” and “higher level non-manual”. The overall hazard ratio for RTW (adjusted for age, sex, geographic area, employer and calendar year) was estimated at 1.32 (95% CI: 1.19–1.46) for “lower non-manual” vs “manual” and at 1.57 (95% CI: 1.40–1.76) for “higher non-manual” vs “manual”.

Vaez et al. [26] investigated sickness absence in the year 2002 among 4891 employees in Sweden who were on long-term sick-leave (> 90 days) due to psychiatric disorders in 1999. The participants were classified into “low, intermediate, or high level of sickness absence (<17, 17–90, and 91–365 days, respectively) or disability pension in 2002”. They were also classified by the skill requirements of their occupations into one of the following four groups:

- High/Intermediate non-manual employees (for example, teachers, physicians, and dentists)
- Assistant non-manual employees (such as technicians, secretaries, and nurses)
- Skilled manual workers (for instance, guardians, and assistant nurses)
- Unskilled manual workers (such as nursing auxiliaries, industry workers, and taxi drivers).

The estimated age and gender adjusted probability of having a low level of sickness absence increased while the probability of having a disability pension decreased monotonically with the skill requirement of the occupation.

### Research hypotheses

This study is first and foremost an exploratory descriptive study. We had, however, a few hypotheses that we wanted to pursue.

H1: The adjusted likelihoods of being i) deceased or recipient of health related welfare benefits and ii) recipient non-health related welfare benefits, compared to being alive and self-reliant at the 1, 3 and 5 year follow-ups depend on which industrial sector the participant belong to prior to the sickness absence.

H2: The adjusted likelihoods of being i) deceased or recipient of health related welfare benefits and ii) recipient non-health related welfare benefits, compared to being alive and self-reliant at the 1, 3 and 5 year follow-ups depend on which job group skill level the participant belong to prior to the sickness absence.

#### A priori expectations

Regarding industries: We expected that the adjusted odds ratios for welfare dependency in the education and training industry would be higher than average, firstly because of results in previous studies (cf. [19, 20]). Secondly because we believe that teaching constitutes a work situation in which it might be especially important not to display any symptoms of mental ill health. In Denmark, the education and training industry is the highest ranking industry for emotional demands at work [27], and it is well established that teaching is associated with extraordinarily high emotional demands [28–30]. It is reasonable to believe that mental health problems may reduce a person’s ability to cope with emotional demands, and that the workers in the education and training industry thereby would be more prone to lose their working ability (especially in teaching work tasks) due to mental health problems than workers in other industry groups with different job demands.

Regarding job group skill levels: We expected that the adjusted odds ratios for welfare dependency would be higher among participants from jobs with low skill requirements than they were among participants from jobs with high skill requirements, firstly because of results in previous studies (cf. [25, 26]). Secondly, because a low skill level often is associated with elevated unemployment rates (cf. [31]). Thirdly, because a low occupational skill level often is associated with increased rates of physical illness (cf. [32]).

## Methods

The statistical analyses of the present study were conducted in accordance with a detailed study protocol that was written and made into a public document (NRCWE file number 2020–10/169, 26th March 2021) before the analysis phase of the project was commenced. The study was, however, not completely blinded. The cohort of the study had previously been included in an examination of RTW during a three-month follow-up period, as a function of age, gender, educational level, employment status and reason for sickness absence [33]. Moreover, a part of the cohort had previously been included in the Danish return-to- work program, which examined RTW during a one-year follow-up period, as a function of an intervention aimed at facilitating RTW [34, 35]. Furthermore, a 10% sample of the cohort had been included in a preliminary effort to develop a cross-validated prediction model for self-reliance 1 year after a participant’s first sickness absence related visit to a jobs and benefits office. The cohort had, however, never been properly examined for RTW as a function of job group and industrial sector, and it had never been examined for the occurrence of health and non-health related welfare benefits one or more years after the first visit to a jobs and benefits office.

### Data material

The material of the present project consists of person-based data from municipal jobs and benefits offices that are linked to data from series of national registers.

#### Data registered at jobs and benefits offices

The Danish Sickness Benefits Act stipulates that a public sickness benefit system should cover long-term sickness absence (> 21 days in 2010–2011, > 30 days in 2012) among employed, unemployed, self-employed and assisting spouses. The change from > 21 to > 30 days was due to a change in the legislation. The system is administered by municipal jobs and benefits offices, which according to the Sickness Benefits Act are committed to follow up and continuously evaluate each sick-listed person’s prognosis of return to the labor force [35].

At the first consultation with the jobs and benefits office, the sick-listed persons were to be classified into one of the following RTW expectation categories:

1. Likely to return to the labor force within 3 months
2. Unlikely to return to the labor force within 3 months but able to participate in activities aimed at facilitating a return
3. Unlikely to return to the labor force within 3 months and unable to participate in activities.

The data to be used in the present project were collected from jobs and benefits offices in 21 (out of 98) Danish municipalities in connection with the above-mentioned Danish return-to-work program [35], which ran from 26 April 2010 to 30 September 2012. The obtained database contains inter alia reason for the sickness absence, the date of the first consultation with the jobs and benefits office and a personal identification number, which enable linkage to data in national registers [36]. The Danish RTW-program included only sick-listed persons in category 2. The database covers, however, also sick-listed persons in category 1 and 3. The sick-listed persons in category 2 were mandated to participate in the Danish RTW-program, where they were allocated to either a control or an intervention group [36]. The sick-listed persons in category 1 and 3 were not eligible for inclusion in the intervention study.

The intervention consisted of three core elements: “(i) establishment of multidisciplinary RTW teams, (ii) introduction of standardized workability assessments and sickness absence management procedures, and (iii) a comprehensive training course for the RTW teams” [35]. All 98 Danish municipalities were invited to submit an application for participation in the program, together with a plan for its implementation. A total of 44 municipalities applied and 21 were selected based on, inter alia, the quality and feasibility of implementation plans (e.g. with respect to availability of resources). The allocation to the intervention or control group was done by means of individual level randomization in three of the selected municipalities and by municipality level cluster randomization among the remaining 17 municipalities [35].

#### Data from national registers

The following registers are used: The Central Person Register (CPR) [37], the Employment Classification Module (ECM) [38] and the Danish Register for Evaluation of Marginalization (DREAM) [39].

The Central Person Register contains, inter alia, information on gender, family type, addresses and dates of birth, death and migrations for every person who is or has been an inhabitant of Denmark sometime between 1968 and present time. The Employment Classification Module contains annual person-information on, inter alia, the socio-economic status, occupation and industry of the inhabitants of Denmark. DREAM contains weekly person-based information on social transfer payments such as maternity/paternity benefits, sickness-absence benefits, unemployment benefits, social security cash benefits, and state educational grants. It has existed since 1991 and covers all inhabitants of Denmark. The weekly benefits data are recorded if the person has been on a benefit for one or more days of the week. However, since only one type of social transfer payment can be registered per week, the above-mentioned social transfer payments are prioritized in the order listed, i.e. maternity/paternity benefits have higher priority than sickness-absence benefits, which in turn have higher priority than unemployment benefits etc. It is not possible to receive sickness absence benefits and unemployment benefits at the same time. People are not entitled to unemployment benefits unless they are well, ready, and available for immediate employment opportunities. If they become too sick to work while receiving unemployment benefits then they may receive sickness absence benefits as a compensation for lost unemployment benefits.

### Study population and inclusion criteria

**Fig. 1:**
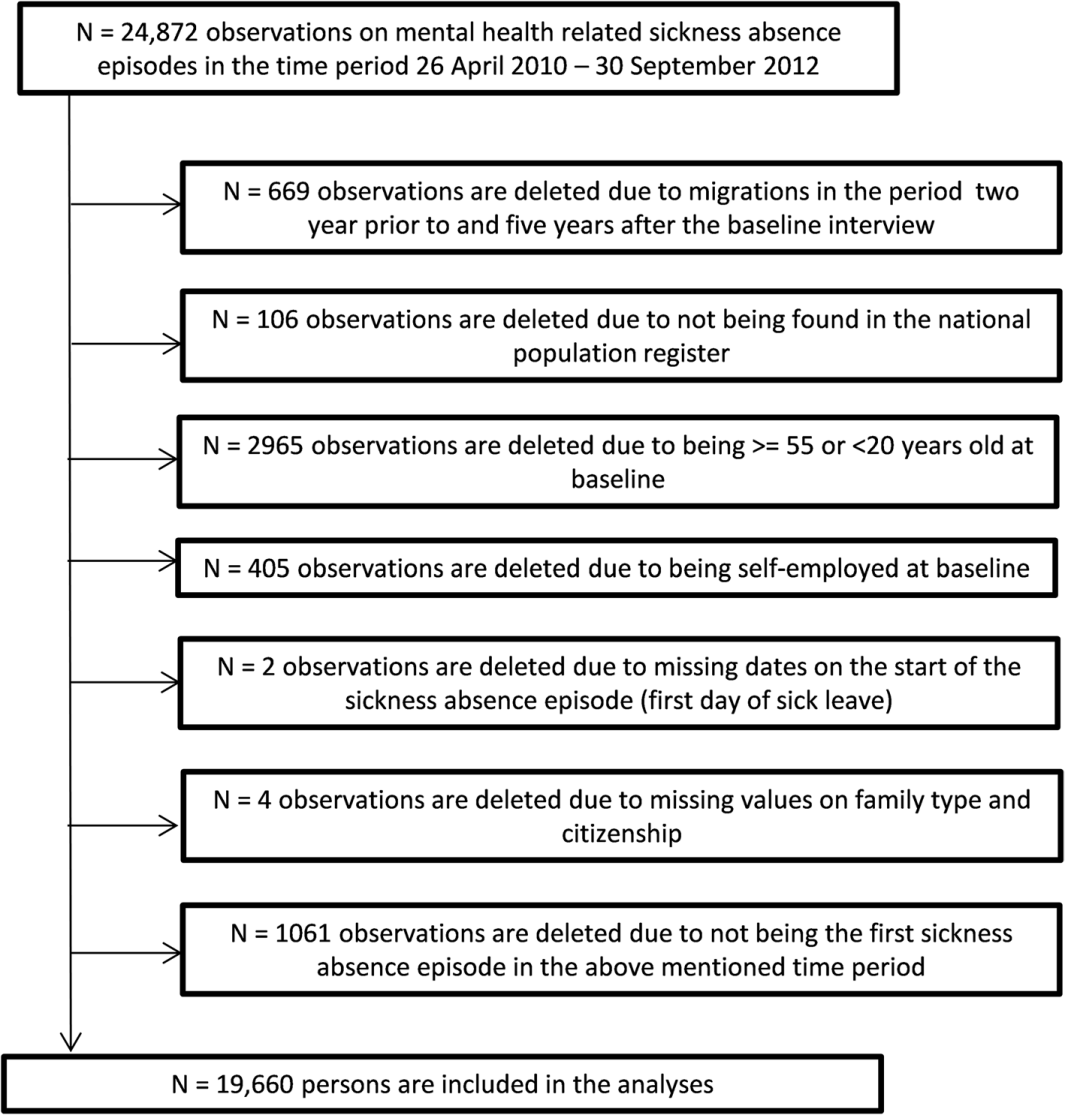
A flow-chart for exclusions of the analysis

The study population consists of all 20–54 year old employed or unemployed people who (according to the jobs and benefits offices in the 21 municipalities of the Danish RTW program) were on long-term sickness-absence due to self-reported depression, anxiety, stress/burnout or mental ill health without further specification, sometime during the period 26 April 2010–30 September 2012. If a person was registered with more than one sickness-absence episode, of the above-mentioned kind during the above-mentioned period, then only the first of the episodes was included in the analyses. To be included in the present study, it was, moreover, required that, from 2 years prior to the concerned sickness absence episode until 5 years after the first visit to the jobs and benefits office, the person did not immigrate or emigrate from one country to another. The participants, could, however, move freely between municipalities within Denmark. National registers enabled us to follow-up on welfare dependency also among the participants who moved to a municipality not included in the study setting. In this way, we minimized the likelihood of attrition bias. In total, 19,660 observations/persons fulfilled the inclusion criteria. A chart of inclusions/exclusions are given in Fig. 1.

### Outcome variable

#### Welfare benefits at follow-up

This is a multinomial variable, which is divided into the following categories:

1. Did not receive any social transfer payments other than holiday allowance (DREAM-code: 121), state educational grants (DREAM-codes: 651, 652, 661) or maternity/paternity leave benefits (DREAM-code: 881)
2. Deceased or recipient of health related social transfer payments (DREAM-codes: 750–818, 890–818)
3. Recipient of other social transfer payments

The social transfer payments are based on registrations in DREAM. The social transfer payments of category two and three are considered adverse outcomes (welfare benefits received due to unfortunate circumstances). Since death is also an adverse (health-related) outcome, it is included in category 2. The odds of being in category 2 can thereby be interpreted as a proxy measure for the odds of being temporarily or permanently out of the labor force due to health issues. Maternity benefits, state educational grants and holiday allowance are not considered adverse outcomes and are therefore included in category 1. In the text that follows, category 1 will be referred to as “self-reliant” while category 3 will be referred to as “recipient of non-health related welfare benefits”.

The outcome is evaluated at a single time point (week), at exactly one, three and 5 years after the baseline interview.

### Covariates

The statistical model includes a series of covariates (independent variables). These covariates are divided into three categories. The first category consists of dummy variables for industrial sectors and job group skill levels. Since the primary aim of the study was to study the effects of these variables, they will be referred to as primary predictors. The remaining covariates were included in order to reduce the possibility of bias in the study of the primary predictors. The statistical analyses provide, however, automatically parameter estimates for all of the included covariates. Some of these covariates are of interest not only as control variables but also as potentially important predictors of welfare dependency after sickness absence due to mental ill health. We therefore decided, as a secondary aim, that if the covariates were of interest as potentially important predictors and it was deemed appropriate to study them as independent predictors within the statistical model and data material of the present study, then we would estimate and report odds ratios for them as well. Since the secondary aim of the study was to study the effects of such variables, they will be referred to as secondary predictors. The remaining covariates will be referred to as control variables. Geographical region at baseline is an example of a variable that in this study serves well as control variable since it may mitigate bias from within-sample geographical differences in e.g. job opportunities. It does, however, not qualify as a secondary predictor; firstly because it is unlikely to be of interest to anyone outside of Denmark and secondly because the data material and statistical model of the study were not designed to study geographical inequalities. A fair comparison of geographical regions requires that the municipalities involved in the study are representative members of the municipalities within their respective regions, which is unlikely to be the case in the present study. Parameter estimates of control variables will not be reported. The classification of the covariates into “primary predictors”, “secondary predictors” and “control variables” was finalized before the analyses were commenced.

#### Primary predictors

##### Industrial sector (last recorded during a 2 year period preceding baseline)

A person’s main industry in a given calendar year is registered annually in the employment classification module. The industrial codes are based on the industrial classification DB07 [40]. In the present study, the industries are divided into the following sectors: Agriculture, forestry, hunting and fishing (DB07-codes: 01.11–03.22); Manufacturing, mining and quarrying (05.10–33.20); Construction (41.10–43.99); Wholesale and retail trade; repair of motor vehicles and motorcycles (45.11–47.99); Transporting and storage (49.10–53.20); Accommodation and food service (55.10–56.30); Public administration (84.11–84.13); Courts and prisons; Police; Fire Departments (84.23–84.25); Education (85.10–85.60); Human health and social work (86.10–88.99); Other; Unstated.

A total of 0.5% of the industry codes were missing (unstated). The following industries were included in the category ‘Other’: Electricity, gas, steam and air-conditioning supply; Water supply, sewerage, waste management and remediation; Information and communication; Financial and insurance activities; Real estate activities; Professional, scientific, technical, administration and support service activities; Other services.

##### Job group skill level (last recorded during a 2 year period preceding baseline)

A person’s main occupation in a given calendar year is registered annually in the employment classification module. The occupations in the present study are divided into the following job group skill levels: Professionals; Technicians and associate professionals; Workers in occupations that require skills at a basic level; Workers in elementary occupations; Workers in occupations without skill requirements, in accordance with the Danish version of the International Standard Classification of Occupations (DISCO) [41].

#### Secondary predictors

The following secondary predictors were included: Self-reported reason for sickness absence (anxiety; depression; mental ill health not otherwise specified (NOS); stress/burnout), gender, age (10-year classes), family type (married or cohabitant with resident children; married or cohabitant without resident children; single with resident children; single without resident children) and employment status (employed; unemployed). Family type refers to the situation at the end of the calendar year preceding baseline. The other predictors refers to the situation at baseline.

In Denmark, long-term sickness-absence does not require an ICD-code diagnosis. Hence, the data registered at the jobs and benefits offices do not contain any ICD-codes on primary causes of sickness absence nor on possible comorbid somatic problems.

#### Control variables

The following control variables were included: Unemployment insurance {Yes (*N* = 17,128); No (*N* = 2532)}, Danish citizenship {Yes (*N* = 18,732); No (*N* = 928)}, calendar year at the start of the sickness absence episode {< 2012 (*N* = 15,876); 2012 (*N* = 3784)}, time passed between the first day of sickness absence and the baseline visit at the jobs and benefits office {≤30 (*N* = 2791); 31–60 (*N* = 12,231); > 60 days (*N* = 4638)}, geographical region at baseline {Capital (*N* = 7365), Zealand (*N* = 2387); Southern Denmark (*N* = 3082); Central Jutland (*N* = 6366) and Northern Jutland (*N* = 460)}, assignment in the Danish RTW-program {intervention group (*N* = 5252); control group (*N* = 3403); not eligible for participation (*N* = 11,005)}, weeks with health related social transfer payments during a two-year period prior to the baseline sickness absence episode {0 (*N* = 11,179); 1–26 (*N* = 6289); > 26 (*N* = 2192)} and ditto for non-health related social transfer payments (other than state educational grants and maternity/paternity leave benefits) {0 (*N* = 11,600); 1–26 (*N* = 4184); > 26 (*N* = 3876)}.

### Statistical analyses

With outcome category 1 (self-reliant) as reference, multinomial logistic regression was used to estimate odds ratios (OR), with 99% confidence interval (CI), for being in outcome category 2 and 3 (“Deceased or recipient of health related welfare benefits” and “Recipient of non-health related welfare benefits”) at 1, 3 and 5 years after the baseline interview, as a function of job group skill level, industrial sector, reason for sickness absence, gender, age, family type and employment status. The effects of job group skill level, industrial sector, gender, age, family type and employment status was estimated in a model that included all of the variables in the sections entitled “Primary predictors”, “Secondary predictors” and “Control variables”. The effects of “reason for sickness absence” was estimated in a model that included all of the above-mentioned variables except for the variable named “assignment in the Danish RTW-program”. Likelihood ratio tests were used to test the null-hypotheses, which stated that the distribution of the outcome categories is independent of job group skill level and industrial sector, respectively. The hypotheses were tested for the status at 1, 3 and 5 years after the baseline interview, respectively. Sub-hypotheses, which stated that the odds-ratio for health and non-health related social transfer payments, respectively, is independent of job group skill level/industrial sector, would be tested if and only if the *P*-value of the parent null-hypothesis test was ≤0.01.

We hold that statistical significance in principle only can be declared in blinded statistical analyses, i.e. in analyses where the hypotheses are completely defined before the researchers have looked at any relation between the concerned exposure and outcome data that are to be used to test them. As mentioned above, the present study is not completely blinded. Hence, *P* > 0.01 would be regarded as” not statistically significant” while *P* ≤ 0.01 would be regarded as “tentatively statistically significant”, where “tentatively” means “subject to further confirmation; not definitely”.

## Results

In total, 19,660 persons (69.8% women) were included in the analyses. The mean ages at baseline were 38.5 (StdDev 8.8) years among the women and 38.8 (StdDev 9.0) years among the men. 23.0% of the included participants were unemployed at baseline. At the 1-year follow-up 54.8% were self-reliant, 22.7% were either deceased or received health related benefits while 22.5% received non-health related benefits. The corresponding percentages at the 3- year follow-up were 61.8, 17.5 and 20.7%. The percentages at the 5-year follow-up were 63.3, 21.0 and 15.7%. A total of 30, 98 and 165 persons were dead at the 1, 3 and 5 year follow-up respectively.

Our first hypothesis (H1) stated that the adjusted likelihoods of receiving health and non-health related welfare benefits at the 1, 3 and 5 year follow-ups depend on which industrial sector the participant belong to prior to the sickness absence. Our results did not lend support to this hypothesis. The *P*-values for “independence of industry” were estimated at 0.0936, 0.7263 and 0.4820 at the 1, 3 and 5 year follow-up, respectively. We had, moreover, hypothesized that the estimated odds for welfare dependency would be significantly higher than average in the education and training industry. All of the odds ratios for the education industry vs. “all other industries combined” were, however, very close to unity with quite narrow confidence interval.

Our second hypothesis (H2) stated that the adjusted likelihoods of receiving health and non-health related welfare benefits at the 1, 3 and 5 year follow-ups depend on which job group skill level the participant belong to prior to the sickness absence. We expected that the adjusted likelihood of receiving welfare benefits at follow-up would be higher among participants from jobs with low skill requirements than among participants from jobs with high skill requirements. The estimated odds ratios for welfare dependency were consistently higher among participants in jobs with the lowest skill requirements than they were among professionals --the group with the highest skill requirements. The results were, however, not statistically significant at our pre-specified significance level 0.01. The *P*-values for “independence of job group skill level” were estimated at 0.0270, 0.3060 and 0.0135 at the 1, 3 and 5-year follow-up respectively.

The estimated odds ratios for each of the primary and secondary predictor variables are given in Tables 1, 2 and 3 for the 1, 3 and 5-year follow-ups, respectively.

**Table 1:** Adjusted odds ratio (OR) with 99% confidence interval (CI) for death or welfare dependency 1 year after first visit to a jobs and benefits office due to mental health related sickness absence, in Denmark, 2010–2012

Predictor variables	Persons	Deceased or recipient of health related welfare benefits			Recipient of non-health related welfare benefits		
		Cases	OR^a^	99% CI	Cases	OR^a^	99% CI
**Industrial sector**^**b**^							
Agriculture, forestry, hunting and fishing	206	60	1.23	0.77–1.96	42	0.82	0.49–1.36
Manufacturing, mining and quarrying	1781	416	1.05	0.88–1.24	402	0.98	0.83–1.17
Construction	1212	286	0.98	0.80–1.21	273	0.91	0.74–1.12
Wholesale and retail trade; repair of motor vehicles	2496	593	1.11	0.96–1.29	576	1.06	0.91–1.23
Transporting and storage	1100	243	0.95	0.77–1.18	259	0.99	0.80–1.23
Accommodation and food service	573	114	0.83	0.61–1.13	164	1.19	0.90–1.57
Public administration	642	131	0.93	0.70–1.24	141	1.06	0.80–1.40
Courts and prisons; Police; Fire Departments	224	56	1.12	0.71–1.76	46	0.97	0.60–1.56
Education	1572	356	1.03	0.85–1.26	330	0.97	0.79–1.18
Human health and social work	5357	1216	0.96	0.85–1.08	1122	0.91	0.81–1.02
Other industries	4396	961	0.97	0.86–1.10	1040	1.09	0.96–1.22
**Job group skill level**							
Workers in elementary occupations	2083	471	1.03	0.83–1.28	530	1.17	0.94–1.45
Workers in occupations that require skills at a basic level	8842	2031	1.06	0.89–1.27	2066	1.12	0.94–1.34
Technicians and associate professionals	4554	1029	1.04	0.86–1.25	889	0.94	0.78–1.14
Professionals	2467	513	1.00	–	510	1.00	–
Employees in occupations with missing skill requirements	1714	422	0.99	0.78–1.26	433	1.03	0.82–1.31
**Reason for the sickness absence**							
Anxiety	667	202	1.98	1.51–2.60	177	1.42	1.08–1.88
Depression	8140	2202	1.75	1.56–1.96	2196	1.52	1.36–1.70
Mental ill health NOS	2197	709	2.11	1.79–2.49	568	1.48	1.25–1.76
Stress/burnout	8656	1353	1.00	–	1487	1.00	–
**Age**							
20–29 years	3850	759	0.46	0.39–0.56	929	0.77	0.64–0.93
30–39 years	7023	1472	0.61	0.51–0.71	1585	0.88	0.74–1.05
40–49 years	6450	1551	0.77	0.65–0.90	1440	0.97	0.82–1.16
50–54 years	2337	684	1.00	–	474	1.00	–
**Family type**							
Married or cohabitant with resident children	8633	1757	0.75	0.66–0.85	1594	0.67	0.59–0.76
Married or cohabitant without resident children	3175	741	0.86	0.74–1.01	663	0.78	0.67–0.91
Single with resident children	2653	647	0.98	0.82–1.16	722	1.04	0.88–1.23
Single without resident children	5199	1321	1.00	–	1449	1.00	–
**Gender**							
Men	5939	1480	1.05	0.93–1.17	1516	1.11	0.99–1.24
Women	13,721	2986	1.00	–	2912	1.00	–
**Employment status at baseline**							
Unemployed	4515	1633	2.20	1.90–2.55	1655	1.89	1.64–2.19
Employed	15,145	2833	1.00	–	2773	1.00	–

^a^The odds ratios of each predictor are mutually adjusted for the other predictors. They are, moreover, adjusted for geographic region, participation in the Danish RTW-program study, Danish citizenship, calendar period and social transfer payments during a two-year period prior to the baseline

^b^The reference (comparison group) for a given industrial sector consist of all other industrial sectors combined

**Table 2:** Adjusted odds ratio (OR) with 99% confidence interval (CI) for death or welfare dependency 3 years after first visit to a jobs and benefits office due to mental health related sickness absence, in Denmark, 2010–2012

Predictor variables	Persons	Deceased or recipient of health related welfare benefits			Recipient of non-health related welfare benefits		
		Cases	OR^a^	99% CI	Cases	OR^a^	99% CI
**Industrial sector**^**b**^							
Agriculture, forestry, hunting and fishing	206	42	0.99	0.60–1.64	38	0.74	0.44–1.23
Manufacturing, mining and quarrying	1781	305	0.94	0.78–1.13	356	0.90	0.76–1.08
Construction	1212	219	0.98	0.78–1.22	264	1.00	0.82–1.23
Wholesale and retail trade; repair of motor vehicles	2496	439	1.02	0.87–1.19	533	1.03	0.89–1.20
Transporting and storage	1100	186	0.93	0.74–1.17	239	1.04	0.84–1.28
Accommodation and food service	573	95	0.93	0.68–1.29	147	1.20	0.91–1.59
Public administration	642	108	1.02	0.76–1.38	121	1.00	0.75–1.33
Courts and prisons; Police; Fire Departments	224	49	1.36	0.86–2.17	48	1.18	0.74–1.89
Education	1572	276	1.07	0.87–1.32	304	0.99	0.80–1.21
Human health and social work	5357	939	1.01	0.89–1.14	1095	1.01	0.90–1.13
Other industries	4396	767	0.99	0.87–1.12	891	0.98	0.86–1.10
**Job group skill level**							
Workers in elementary occupations	2083	395	1.23	0.98–1.55	442	1.05	0.84–1.31
Workers in occupations that require skills at a basic level	8842	1593	1.20	0.99–1.46	1872	1.08	0.90–1.29
Technicians and associate professionals	4554	754	1.09	0.89–1.34	897	1.06	0.87–1.28
Professionals	2467	377	1.00	–	459	1.00	–
Employees in occupations with missing skill requirements	1714	331	1.18	0.92–1.51	398	1.04	0.82–1.32
**Reason for the sickness absence**							
Anxiety	667	156	1.97	1.49–2.60	173	1.68	1.28–2.20
Depression	8140	1609	1.54	1.37–1.75	2139	1.69	1.51–1.90
Mental ill health NOS	2197	563	2.09	1.76–2.48	558	1.66	1.40–1.97
Stress/burnout	8656	1122	1.00	–	1198	1.00	–
**Age**							
20–29 years	3850	489	0.35	0.29–0.42	958	0.85	0.71–1.03
30–39 years	7023	1115	0.52	0.44–0.61	1376	0.79	0.66–0.94
40–49 years	6450	1242	0.68	0.58–0.81	1293	0.90	0.75–1.08
50–54 years	2337	604	1.00	–	441	1.00	–
**Family type**							
Married or cohabitant with resident children	8633	1366	0.72	0.63–0.83	1372	0.63	0.55–0.72
Married or cohabitant without resident children	3175	603	0.86	0.73–1.01	577	0.70	0.60–0.82
Single with resident children	2653	470	0.90	0.75–1.09	732	1.18	1.00–1.39
Single without resident children	5199	1011	1.00	–	1387	1.00	–
**Gender**							
Men	5939	1151	1.07	0.95–1.20	1406	1.10	0.98–1.23
Women	13,721	2299	1.00	–	2662	1.00	–
**Employment status at baseline**							
Unemployed	4515	1077	1.39	1.18–1.62	1596	1.57	1.37–1.81
Employed	15,145	2373	1.00	–	2472	1.00	–

^a^The odds ratios of each predictor are mutually adjusted for the other predictors. They are, moreover, adjusted for geographic region, participation in the Danish RTW-program study, Danish citizenship, calendar period and social transfer payments during a two-year period prior to the baseline

^b^ The reference (comparison group) for a given industrial sector consist of all other industrial sectors combined

**Table 3:** Adjusted odds ratio (OR) with 99% confidence interval (CI) for death or welfare dependency 5 years after first visit to a jobs and benefits office due to mental health related sickness absence, in Denmark, 2010–2012

Predictor variables	Persons	Deceased or recipient of health related welfare benefits			Recipient of non-health related welfare benefits		
		Cases	OR^a^	99% CI	Cases	OR^a^	99% CI
**Industrial sector**^**b**^							
Agriculture, forestry, hunting and fishing	206	53	1.24	0.78–1.98	37	1.12	0.67–1.89
Manufacturing, mining and quarrying	1781	378	0.99	0.83–1.18	256	0.84	0.69–1.03
Construction	1212	280	1.09	0.89–1.34	199	1.01	0.81–1.27
Wholesale and retail trade; repair of motor vehicles	2496	510	0.95	0.81–1.10	392	0.95	0.81–1.12
Transporting and storage	1100	230	0.99	0.80–1.23	186	1.07	0.85–1.35
Accommodation and food service	573	111	0.80	0.59–1.09	92	0.84	0.61–1.16
Public administration	642	140	1.13	0.86–1.49	85	0.97	0.70–1.34
Courts and prisons; Police; Fire Departments	224	52	1.10	0.70–1.73	36	1.11	0.66–1.85
Education	1572	312	0.99	0.81–1.21	229	0.99	0.79–1.23
Human health and social work	5357	1158	1.06	0.94–1.18	821	1.02	0.90–1.16
Other industries	4396	882	0.94	0.83–1.06	722	1.08	0.95–1.24
**Job group skill level**							
Workers in elementary occupations	2083	465	1.27	1.02–1.58	354	1.22	0.96–1.55
Workers in occupations that require skills at a basic level	8842	1891	1.22	1.02–1.46	1454	1.22	1.00–1.49
Technicians and associate professionals	4554	932	1.14	0.94–1.38	635	1.05	0.85–1.31
Professionals	2467	441	1.00	–	333	1.00	–
Employees in occupations with missing skill requirements	1714	404	1.27	1.01–1.61	303	1.16	0.90–1.51
**Reason for the sickness absence**							
Anxiety	667	155	1.59	1.21–2.09	137	1.58	1.18–2.12
Depression	8140	2011	1.69	1.51–1.90	1595	1.66	1.46–1.89
Mental ill health NOS	2197	647	2.13	1.81–2.50	451	1.78	1.48–2.14
Stress/burnout	8656	1320	1.00	–	896	1.00	–
**Age**							
20–29 years	3850	574	0.31	0.25–0.37	720	0.77	0.63–0.95
30–39 years	7023	1320	0.48	0.41–0.56	1059	0.79	0.65–0.96
40–49 years	6450	1522	0.68	0.58–0.79	969	0.89	0.73–1.08
50–54 years	2337	717	1.00	–	331	1.00	–
**Family type**							
Married or cohabitant with resident children	8633	1695	0.82	0.72–0.93	991	0.57	0.50–0.66
Married or cohabitant without resident children	3175	700	0.90	0.76–1.05	419	0.63	0.53–0.75
Single with resident children	2653	605	0.99	0.84–1.18	538	1.04	0.87–1.24
Single without resident children	5199	1133	1.00	–	1131	1.00	–
**Gender**							
Men	5939	1312	0.97	0.87–1.09	1093	1.07	0.95–1.21
Women	13,721	2821	1.00	–	1986	1.00	–
**Employment status at baseline**							
Unemployed	4515	1254	1.22	1.05–1.42	1243	1.61	1.38–1.88
Employed	15,145	2879	1.00	–	1836	1.00	–

^a^The odds ratios of each predictor are mutually adjusted for the other predictors. They are, moreover, adjusted for geographic region, participation in the Danish RTW-program study, Danish citizenship, calendar period and social transfer payments during a two-year period prior to the baseline

^b^ The reference (comparison group) for a given industrial sector consist of all other industrial sectors combined

As shown in the tables, the estimated odds ratios were quite independent of gender. They increased, however, markedly with age and the age effect was especially pronounced for the health-related welfare benefits. The tables indicate, moreover, that the odds for welfare dependencies were substantially lower among participants with self-reported stress or burnout compared to participants reporting anxiety, depression or mental ill health not otherwise specified (NOS), as the reason for sickness absence. The effect of reason for sickness absence seems to be stronger for death or health related benefits than it is for non-health-related benefits.

The odds ratios for welfare dependency at follow-up as a function of family type tended to be lower among the married/cohabitants than they were among the single participants. The cohabitation effect tended to be especially strong for non-health-related welfare benefits (Tables 1, 2 and 3).

Tables 1, 2 and 3 show, moreover, that welfare dependency at follow-up were highly dependent on baseline employment status with increased odds ratios both for the health and non-related benefits among the participants who were unemployed at baseline. The odds ratios were especially high at the 1-year follow-up.

## Discussion

We tested two pre-specified hypotheses, one of them (H1) concerned industrial sectors while the other (H2) concerned job group skill levels. None of the hypothesis tests reached statistical significance. Hence, we cannot reject the null-hypotheses that welfare dependency after long-term sickness absence due to mental health problems is independent of the industrial sector and the job group skill level the participant belonged to prior to the sickness absence.

In the introduction, we mentioned that two large studies had found significant effects of industrial sector on the duration of work absence among persons who were sick-listed due to mental health problems, and that the education industry was associated with the longest work absence in both of the studies [19, 20]. The respective study populations came from Australia [19] and the Netherlands [20]. The results of these previous studies do not align well with the results of the present study. In the study from the Netherlands, Koopmans et al. [20] noted that the mean age of employees in the educational and public sectors was higher than average and suggested that this was a possible explanation for the longer work absences in these industries. They did not adjust their analyses for age but concluded that further research was needed to examine this possible explanation. The analyses of the present study lends support to this explanation, since no increased odds was found in the educational sector after adjustment for age. Another possible explanation for the disagreement between the results of Koopmans et al. and the results of the present study is that the endpoint in Koopmans et al. was return to the same job the person held at the start of the sickness absence episode. Absences that ended, because the employee resigned were censored. In the present study, we did not censor participants who decreased their welfare dependency by finding a new job in another industrial sector. A possible reason for the disagreement between the present study and the Australian study [19] is that the latter only included work-related mental health conditions.

The socioeconomic status (SES) of employees are usually defined by the skill requirements of the job they are holding and is sometimes simplified and aggregated into blue- vs. white-collar or manual vs. non-manual workers. Recent reviews have concluded that the association between SES and RTW among employees on sick-leave due to mental health problems is inconclusive (cf. [6, 8]). We found five studies, which examined RTW-related outcomes as a function of SES divided into two or more job group skill levels, three from Finland [8, 23, 25], one from Sweden [26] and one from Canada [21]. The studies from Finland and Sweden reported a positive and statistically significant association between SES and RTW (the higher the skill level the higher the rates of RTW) while the study from Canada reported a statistically significant association in the opposite direction. In the present study, the association between job group skill level and welfare dependency at follow-up was not statistically significant. The results pointed, however, in a direction that aligns with the results obtained in the studies from Finland and Sweden.

It has previously been reported that anxiety and depressive disorders are associated with a longer time until return to work compared with adjustment disorders [42], stress/burnout related disorders [24] and other common mental disorder [43]. The previous studies were, however, small and the statistical precisions of their estimated associations were thereby quite low. In the present study, the estimated odds of health as well as non-health related welfare benefits at the one, three and five-year follow-ups were substantially lower among participants with stress/burnout than they were among the participants with anxiety and depression. The present study thereby strengthens the previous finding that anxiety and depressive disorders are associated with a less favorable RTW outcome than stress-related disorders. Anxiety and depressive disorders occur often without an obvious trigger or stressful event. Stress and burnout, on the other hand, tend to have a known environmental cause. It is reasonable to believe that a mental health problem with a known cause is easier to handle than a mental health problem with an unknown cause, and this may be the reason for the participants with stress/burnout at baseline to have lower odds for welfare dependency at follow-up compared with the participants with anxiety or depression at baseline.

To our knowledge, the present study is the only one that have examined health and non-health related welfare dependencies after sickness absence due to mental ill health as a function of marital status/cohabitation with and without resident children. The outcomes of the present study tended to be more favorable among married/cohabitants than among single participants regardless of resident children. The estimated effects of cohabitation were typically stronger for non-health related welfare benefits. Previous studies on RTW as a function of marital status/cohabitation [43, 44] or family type [45] have been too small to impart any meaningful information.

Recent literature reviews provide strong evidence for a negative association between age and RTW [6, 7, 12], which is in line with the results of the present study. The gender effect appears to be negligible in the present study and previous evidence of an association between gender and RTW is inconsistent (cf. [6, 7]).

As mentioned in the introduction, Engstrom et al. [22] did not find any statistically significant associations between job groups and health-related welfare dependency at 2 and 3 years after long-term sickness absence due stress-related psychiatric diagnoses, in the Swedish labor force. Their conclusion was that “individual labor market position, as occupation, employer, branch etc. seems to be less important than expected in explaining return to work from sickness absence due to stress-related psychiatric disorders”. They found, however, that unemployment at baseline was a strong predictor of health-related welfare dependency at the two and three-year follow-ups. The results of the present study aligns very well with the results by Engstrom et al. [22].

### Strengths, weaknesses and limitations

Since a study protocol was written (and made into a public document) before the analyses of the study were commenced, the risk of hindsight bias was reduced. The risk of bias due to missing follow-up data was minimized, since the participants who migrated during the study period were excluded and the outcomes of the study were ascertained through records in national registers, which covers all residents of Denmark. The study was, moreover, free from volunteer bias since register studies of the present type may be conducted without informed consent by the participants. Another advantage of the study is that the data allowed us to differentiate between health and non-health related welfare benefits. The study was further strengthened by its size and its prospective design.

A major drawback of the study is that the blinding had been compromised. It should, however, be noted that none of the odds ratios that we were to estimate had been estimated before and that it is the odds ratios that constitute the essence of our study. Another drawback is that the reasons for sickness absence were based on self-reports rather than clinical diagnoses (cf. [46]).

Since register studies of the present type may be conducted without informed consent by the participants, we can rule out individual-level volunteer bias. We can, however, not rule out the possibility of volunteer bias at the municipality level. The data were collected in connection with an RTW intervention study that took place in a selected set of Danish municipalities. A total of 44 out of 98 Danish municipalities had volunteered to participate in the study and 21 of these were selected for inclusion, by the research team. The estimated odds of the present study are thereby open to volunteer bias as well as researcher selection bias, at the municipality level. It is, moreover, possible that the presence of the researchers may have influenced behaviors and decisions of the case managers in the concerned jobs and benefits offices. We have, however, not found any reasons to believe in a differential bias among the exposure categories of the examined predictors.

It should also be noted that the odds of receiving welfare benefits depend on the rules and regulations of the concerned welfare benefit systems and that such systems varies between nations and time periods. In Denmark, you can only receive unemployment benefits for a maximum consecutive period of 2 years and you can only receive sickness absence benefits for a maximum consecutive period of 1 year. Moreover, you are not entitled to cash benefits unless you (as well as your spouse or cohabitant partner) are destitute and you are not entitled to unemployment benefits unless you have an unemployment insurance. Being self-reliant is therefore not equivalent to being gainfully employed. In the present study, it can also mean that one is living on private means or that one is supported by a spouse or cohabitant partner. Another feature of the Danish welfare systems is that the requirements to be eligible for the various benefits are independent of industry and job group skill level. The null-findings of the present study with respect to effects of industry and job group skill levels on welfare dependency after sick-leave due to mental health issues will probably not hold good in systems where the conditions and coverage of unemployment and sickness benefits insurances depend on private insurances that are provided by the employer.

## Conclusions

As mentioned in the introduction, preventive efforts are needed to reduce the high costs (for the concerned individuals as well as for the society at large) of sickness absence due to mental ill health. The present study examined industry and job group skill level as potential predictors of welfare dependency after health related sickness absence, in the general population of Denmark, but did not find any significant associations. Our study suggests, however, that the vulnerability lies in population groups characterized by unemployment, older age, being single and being on sick leave due to self-reported anxiety or depression versus stress/burnout. Our study thereby suggests that intervention strategies aimed at facilitating RTW after sickness absence due to mental ill health might have a greater chance of success if they address the condition itself (e.g. through health promotion and psychological treatment) than they do if they focus on work factors, such as industrial sector and job group skill level. It is possible that similar effects of age, family type, gender, unemployment and self-reported reason for sickness absence on the odds of receiving welfare benefits after sickness absence due to mental health problems will be found in other nations with similar welfare benefits systems. We have not found any reasons to believe otherwise. The generalizability of the findings can, however, not be ascertained until similar studies have been conducted in other nations.

## Data Availability

The data that support the findings of this study are available from Statistics Denmark, but restrictions apply to the availability of these data, which were used under license for the current study, and so are not publicly available. Data are however available from the authors upon reasonable request and with permission of Statistics Denmark.

## Abbreviations

CI: Confidence interval
CPR: The Central Person Register
DISCO: Danish version of the International Standard Classification of Occupations
DREAM: Danish Register for Evaluation of Marginalization
ECM: Employment Classification Module
GDP: Gross Domestic Products
ICD: International Classification of Diseases
MHP: Mental health problems
NOS: Not otherwise specified
OR: Odds ratios
RTW: Return to work
StdDev: Standard deviation
vs.: Versus
